# Recruiters to randomised trials can be trained to facilitate recruitment and informed consent by exploring patients’ treatment preferences

**DOI:** 10.1186/1745-6215-14-S1-O63

**Published:** 2013-11-29

**Authors:** Nicola Mills, Jane Blazeby, Freddie Hamdy, David Neal, Bruce Campbell, Jenny Donovan

**Affiliations:** 1 University of Bristol, Bristol, UK; 2 University of Oxford, Oxford, UK; 3 University of Cambridge, Cambridge, UK; 4 University of Exeter, Exeter, UK

## Background

Patient preferences for treatments are often cited as barriers to recruitment in randomised controlled trials (RCTs) but little is known about how to approach this issue in the context of trial recruitment. We investigated how recruitment staff reacted to patients’ treatment preferences within three different pragmatic RCTs.

## Methods

Audio-recordings of 85 RCT recruitment appointments with 69 participants in three UK multicentre RCTs were analysed using content and thematic analysis. Recruiters’ responses to treatment preferences were assessed in one RCT (ProtecT), where training on exploring preferences was given, and compared with two other RCTs where this type of specific training was not given.

## Results

Recruiters elicited treatment preferences similarly in all RCTs, but responses to them differed substantially. In the ProtecT RCT, recruiters explored participants’ preferences at length in four key ways: eliciting and acknowledging the preference rationale, balancing treatment views, empathising with the situation, and emphasising the need to keep an open mind and consider all treatments. Conversely in the other RCTs, treatment preferences were usually accepted by recruiters with little discussion and few patients randomised.

## Conclusion

Recruiters can be trained to elicit and address patients’ treatment preferences enabling those who may not have considered trial participation to do so. Training interventions for recruiters are required to facilitate recruitment and informed consent.

